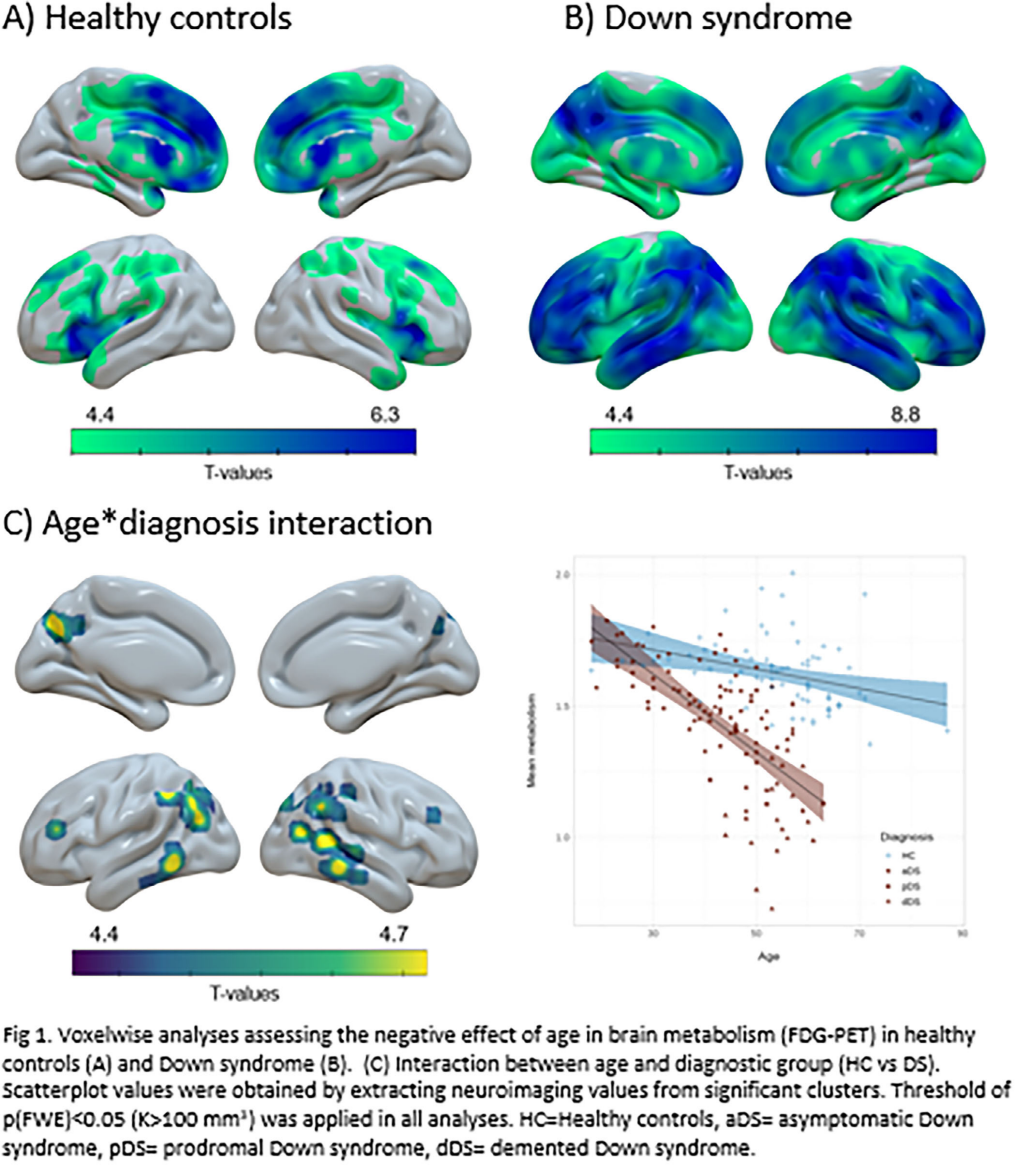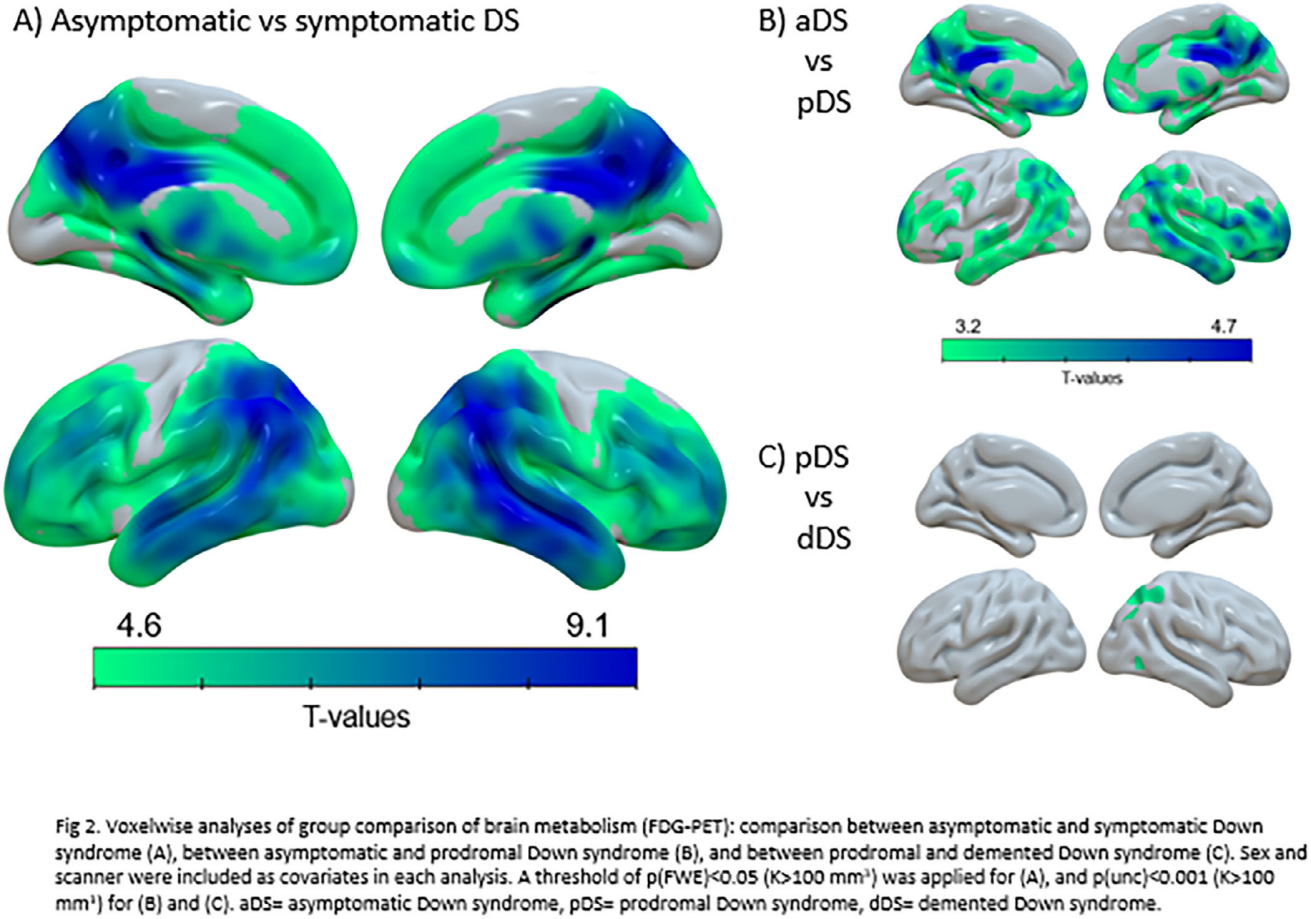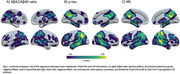# Regional brain metabolism across the Alzheimer's Disease continuum in Down Syndrome

**DOI:** 10.1002/alz.092967

**Published:** 2025-01-09

**Authors:** José Enrique Arriola‐Infante, Alejandra O. Morcillo‐Nieto, Sara E Zsadanyi, María Franquesa‐Mullerat, Lídia Vaqué‐Alcázar, Mateus Rozalem Aranha, Javier Arranz, Íñigo Rodríguez‐Baz, Lucía Maure‐Blesa, Laura Videla, Isabel Barroeta, Laura Del Hoyo, Bessy Benejam, Susana Fernandez, Aida Sanjuan Hernandez, Sandra Giménez, Daniel Alcolea, Olivia Belbin, Albert Flotats, Valle Camacho, Alberto Lleo, Maria Carmona‐Iragui, Juan Fortea, Alexandre Bejanin

**Affiliations:** ^1^ Sant Pau Memory Unit, Hospital de la Santa Creu i Sant Pau, Biomedical Research Institute Sant Pau, Universitat Autònoma de Barcelona, Barcelona, Barcelona Spain; ^2^ Sant Pau Memory Unit, Hospital de la Santa Creu i Sant Pau, Biomedical Research Institute Sant Pau, Universitat Autònoma de Barcelona, Barcelona Spain; ^3^ Sant Pau Memory Unit, Department of Neurology, Hospital de la Santa Creu i Sant Pau, Biomedical Research Institute Sant Pau ‐ Universitat Autònoma de Barcelona, Barcelona Spain; ^4^ Department of Medicine, Faculty of Medicine and Health Sciences, Institute of Neurosciences, University of Barcelona, Barcelona, Spain. Institut d’Investigacions Biomèdiques August Pi i Sunyer (IDIBAPS), Barcelona Spain; ^5^ Hospital de la Santa Creu i Sant Pau ‐ Biomedical Research Institute Sant Pau ‐ Autonomous University of Barcelona, Barcelona, Catalonia Spain; ^6^ Center of Biomedical Investigation Network for Neurodegenerative Diseases (CIBERNED), Madrid Spain; ^7^ Barcelona Down Medical Center, Fundació Catalana Síndrome de Down, Barcelona Spain; ^8^ Center for Biomedical Investigation Network for Neurodegenerative Diseases (CIBERNED), Madrid Spain; ^9^ Multidisciplinary Sleep unit. Hospital de la Santa Creu i Sant Pau, Institut d'Investigació Biomèdica Sant Pau (IIB SANT PAU), Barcelona Spain; ^10^ Hospital de la Santa Creu i Sant Pau ‐ Biomedical Research Institute Sant Pau ‐ Autonomous University of Barcelona, Barcelona Spain; ^11^ Nuclear Medicine Department, Hospital de la Santa Creu i Sant Pau, Barcelona Spain; ^12^ Sant Pau Memory Unit, Hospital de la Santa Creu i Sant Pau ‐ Biomedical Research Institute Sant Pau ‐ Universitat Autònoma de Barcelona, Barcelona Spain

## Abstract

**Background:**

To date, limited data exist concerning the utility of FDG‐PET in detecting Alzheimer's Disease (AD) in Down Syndrome (DS). Yet, sensitive biomarkers for neurodegeneration are essential in this population genetically predisposed for AD. Therefore, we aimed at characterizing the effect of age, disease stage and AD pathology on brain metabolism in a large cohort of adults with DS.

**Method:**

Cross‐sectional study with 72 controls (HC) and 100 participants with DS (63 asymptomatic [aDS], 13 prodromal AD [pDS], 24 AD dementia [dDS]) from the Down‐Alzheimer Barcelona Neuroimaging Initiative, undergoing 3T‐MRI, ^18^F‐FDG‐PET, lumbar puncture, and clinical assessment. ^18^F‐FDG‐PET scans were normalized using MRI data and scaled using the pons. CSF biomarkers were assessed utilizing Lumipulse (Aβ42/Aβ40 ratio and phosphorylated tau [p‐tau]) and ELISA (neurofilament light chain [NfL]) methods. Voxelwise analyses in SPM12 examined i) the effect of age, ii) AD clinical stage and iii) relationships with CSF biomarkers on brain metabolism.

**Result:**

In HCs, brain metabolism decreased with age mainly in the frontal lobe, while in DS we observed a more distributed pattern, predominating in temporoparietal regions (Figure 1A‐B). The interaction indicated greater metabolic loss with age in DS compared to HC in lateral temporoparietal regions (Figure 1C). Compared to aDS, symptomatic DS exhibited lower metabolism in most regions, with the strongest effect in medial parietal and lateral temporoparietal structures (Figure 2). Additional comparisons revealed a clear decreased metabolism in posterior cingulate and angular gyrus in pDS vs aDS, and a more subtle metabolic loss in posterior lateral parietal areas in dDS compared to pDS. Associations between metabolism and CSF biomarkers were found in brain regions showing hypometabolism in symptomatic AD, and were most significant for NfL (Figure 3).

**Conclusion:**

The age‐related decrease in brain metabolism was more pronounced in DS compared to HCs and likely reflects AD processes. The pattern of hypometabolism at the symptomatic stage predominated in temporoparietal regions and strongly resembles the pattern observed in sporadic AD, further emphasizing similarities between genetic and sporadic AD forms. The hypometabolism in parietal structures at the prodromal stage demonstrated ^18^F‐FDG‐PET´s sensitivity as biomarker for early AD diagnosis in DS.